# Corticolimbic Modulation via Intermittent Theta Burst Stimulation as a Novel Treatment for Functional Movement Disorder: A Proof-of-Concept Study

**DOI:** 10.3390/brainsci11060791

**Published:** 2021-06-15

**Authors:** Primavera A. Spagnolo, Jacob Parker, Silvina Horovitz, Mark Hallett

**Affiliations:** 1 Mary Horrigan Connors Center for Women’s Health & Gender Biology, Department of Psychiatry, Brigham and Women Hospital, Thorn Building, 75 Francis St, Boston, MA 02115, USA; 2 Harvard Medical School, Boston, MA 02115, USA; 3 Human Motor Control Section, Medical Neurology Branch, National Institute of Neurological Disorders and Stroke, National Institutes of Health, Building 10, Room 7D37, 10 Center Drive, Bethesda, MD 20892, USA; jacob.parker@nih.gov (J.P.); silvina.horovitz@nih.gov (S.H.)

**Keywords:** functional movement disorders, neuromodulation, intermittent theta burst stimulation, amygdala, corticolimbic connectivity, valence

## Abstract

Neuroimaging studies suggest that corticolimbic dysfunctions, including increased amygdala reactivity to emotional stimuli and heightened fronto-amygdala coupling, play a central role in the pathophysiology of functional movement disorders (FMD). Transcranial magnetic stimulation (TMS) has the potential to probe and modulate brain networks implicated in neuropsychiatric disorders, including FMD. Therefore, the objective of this proof-of-concept study was to investigate the safety, tolerability and preliminary efficacy of fronto-amygdala neuromodulation via targeted left prefrontal intermittent theta burst stimulation (iTBS) on brain and behavioral manifestations of FMD. Six subjects with a clinically defined diagnosis of FMD received three open-label iTBS sessions per day for two consecutive study visits. Safety and tolerability were assessed throughout the trial. Amygdala reactivity to emotionally valenced stimuli presented during an fMRI task and fronto-amygdala connectivity at rest were evaluated at baseline and after each stimulation visit, together with subjective levels of arousal and valence in response to affective stimuli. The FMD symptom severity was assessed at baseline, during treatment and 24 h after the last iTBS session. Multiple doses of iTBS were well-tolerated by all participants. Intermittent TBS significantly decreased fronto-amygdala connectivity and influenced amygdala reactivity to emotional stimuli. These neurocircuitry changes were associated to a marked reduction in FMD symptom severity. Corticolimbic modulation via iTBS represents a promising treatment for FMD that warrants additional research.

## 1. Introduction

Functional movement disorders (FMD), part of the wide spectrum of functional neurological disorders, represent one of the most common conditions encountered by neurologists and neuropsychiatrists today [1]. Despite the high prevalence and the considerable individual and societal burdens associated with these disorders, their etiopathogenesis is still elusive and only a few, evidence-based treatments are currently available for patients with FMD [2].

In recent years, however, there have been substantial advances in the pathophysiologic understanding of these disorders, which have critically impacted the therapeutic management of FMD [3]. In particular, functional neuroimaging studies have provided extensive evidence of alterations in activity and connectivity in brain networks mediating motor functions, cognitive control, emotion processing, and perceptual awareness [4,5]. Among these alterations, cortico-limbic dysfunctions appear to play a central role in the pathophysiology of FMD. Indeed, increased amygdala reactivity to emotionally valenced stimuli has been repeatedly reported in patients with FMD, together with heightened amygdala coupling to motor and cognitive control regions, and abnormal activation of several prefrontal regions during emotion processing tasks [6,7,8]. These findings suggest that these disorders are characterized by an impairment in bottom-up and top-down mechanisms in cortico-limbic circuits during emotion processing, which may exert an abnormal influence on motor systems [9,10].

This emerging disease model of FMD as a disorder of impaired neurocircuitry provides a robust rationale for the development of neurobiologically-based treatments aimed at targeting and remodeling dysregulated brain circuits. This hypothesis is supported by preliminary findings, showing that the efficacy of motor rehabilitation and cognitive behavioral therapy in reducing functional motor symptoms is associated with changes in brain regions and networks implicated in FMD [11,12].

A further promising intervention garnering significant attention is transcranial magnetic stimulation (TMS). This non-invasive brain stimulation technique alters cortical excitability in the targeted cortex as well as in its downstream interconnections via transcranial delivery of magnetic pulses at different intensities and frequencies. By applying a patterned, repetitive sequence of TMS (rTMS), temporally sustained changes in neuronal firing can be achieved, causing changes in brain functions and behavior [13]. For instance, rTMS can affect cognitive processes, such as attentional control, as well as response to rewards and affective stimuli [14]. These effects could be long-lasting, following multiple stimulation sessions, and are thought to reflect the ability of rTMS to enhance synaptic plasticity in a dose-dependent manner [15]. This mechanism is thought to underlie the therapeutic effects of rTMS, which is currently approved by the Food and Drug Administration for the treatment of depression, OCD and nicotine addiction.

In the field of FMD, the use of rTMS as a therapeutic tool is still in its infancy. The published literature includes case report series and a small number of sham-controlled trials, characterized by a substantial variability in design and stimulation parameters [16,17,18,19]. Importantly, these studies evaluated rTMS effects on clinical and behavioral outcomes of FMD; it is unknown whether rTMS induces neuromodulatory and neuroplastic changes in relevant brain networks, which may underlie the observed clinical effects.

To begin addressing these questions, we conducted a pilot, proof-of-concept study in a sample of patients with hyperkinetic FMD with the aim to (i) assess the safety and feasibility of multiple sessions of an accelerated intermittent theta burst stimulation (iTBS) protocol (3600 pulses); and (ii) uncover the effects of prefrontal iTBS on FMD-associated cortico-limbic dysfunctions (amygdala hyperreactivity to emotional stimuli and increased fronto-amygdala connectivity).

Targeting the left dorsolateral prefrontal cortex (DLPFC) via excitatory rTMS and iTBS is shown to improve emotion processing in both healthy and patient populations by inhibiting negative bias and increasing response for positive stimuli [20,21,22], while right-sided prefrontal stimulation is associated with increased right amygdala activation and enhanced attentional allocation to threatening stimuli [23,24]. In addition to modulating amygdala reactivity during emotion processing, left prefrontal iTBS is also shown to decrease functional connectivity between DLPFC and limbic regions [25,26]. This specific mechanism may further support the therapeutic use of left prefrontal excitatory stimulation in FMD patients, given that individuals with FMD compared to controls exhibit heightened connectivity between the amygdala and DLPFC [8]. Several studies in psychiatric populations found an association between reduction in abnormal hyperconnectivity patterns via TMS and iTBS and improvement in symptom severity [27,28].

On the basis of these observations, we hypothesized that an accelerated protocol of left DLPFC iTBS in individuals with FMD would normalize fronto-amygdala hyperconnectivity and promote a dissociative pattern of amygdala response to negative and positive emotional stimuli presented during functional magnetic resonance imaging (fMRI) sessions. To obtain further insights into the mechanisms of action of iTBS, we also explored the effects of stimulation on subjective valence and arousal levels in response to affective stimuli and on FMD symptomatology.

## 2. Materials and Methods

### 2.1. Participants

Twenty-five patients with clinically definite FMD as diagnosed by at least two movement disorder specialists, using Fahn and Williams criteria [29], were recruited through the Human Motor Control Clinic at the NIH between February 2018 and May 2019. Participants belonged to a larger ongoing study investigating the clinical and neurobiological correlates of FMD. After the first phone screen, 8 subjects declined to participate due to scheduling conflicts and 10 subjects did not qualify due to contraindications to TMS or MRI. Details on inclusion and exclusion criteria are provided in the Supplemental Material. The remaining 7 participants underwent an eligibility screening during the first visit, following which one subject was excluded, given a prior diagnosis of tinnitus and hearing loss.

We performed the experiments in agreement with relevant guidelines and regulations [30,31]. The NIH Institutional Review Board approved the study. All participants provided written informed consent.

### 2.2. Study Design

**Figure 1:**
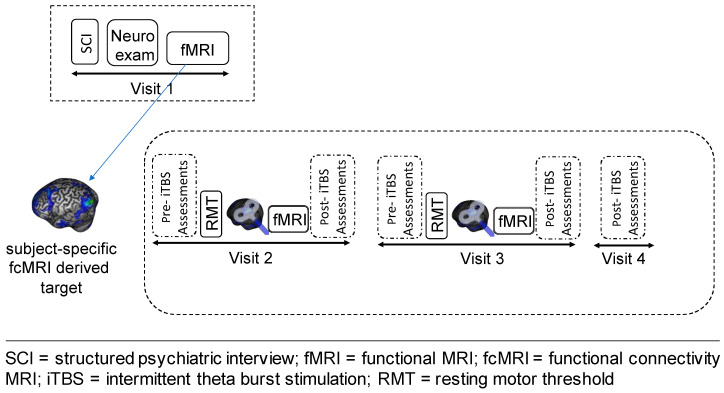
Schematic representation of the study design.

This was a proof-of-concept, feasibility, open-label study, which included four outpatient visits (Figure 1). On visit 1 (V1), we evaluated study eligibility, obtained informed consent and performed a comprehensive assessment of the clinical phenotype of study subjects through a neurological exam, a semi-structured clinical interview for psychiatric diagnosis (SCID-IV-R) [32] and a battery of self-report and clinician-rated scales and questionnaires (for details, see Appendix A). Further, participants underwent a baseline MRI scanning session to acquire T1-weighted, resting-state functional connectivity (rsFC) and task-based data. In addition to evaluating DLPFC-amygdala FC prior to the stimulation, the rsFC data were used to identify the cortical iTBS target within the left DLPFC that was functionally connected to the left amygdala, as described below.

Following the MRI session, we measured subjective levels of arousal and valence in response to affective face stimuli, using the self-assessment Manikin (SAM) [33]. The SAM is a pictorial rating scale that directly measures the affective dimension of valence and arousal in response to emotionally valenced stimuli. A complete description of this procedure can be found in Appendix A.

After a period of 1–2 weeks from visit 1, subjects participated in three consecutive outpatient visits, which were separated by a 24 h interval. During visit 2 and 3 (V2 and V3), they received a block of three sessions of iTBS to the individualized left DLPFC target mapped, using an online, MRI-guided neuronavigation system. Approximately 15–20 min after the last iTBS session, participants underwent an fMRI scan (task-based and rsfMRI), and then provided valence and arousal levels in response to emotional stimuli, as described above.

We also collected several additional clinical and subjective measures, including the severity and frequency of functional motor symptoms, which were assessed at 6 different timepoints (V1, V2: pre- and post-iTBS; V3: pre- and post-iTBS, and at V4) using a modified version of the Simplified- Functional Movement Disorders Rating Scale (S-FMDRS) [34]. Furthermore, following each stimulation block, we administered the iTBS monitoring questionnaire, an internally generated 13-item interview-based yes/no questionnaire assessing side effects of iTBS (e.g., headaches, nausea, seizure), the Positive and Negative Affect Scale [35] and the Young Mania Rating Scale [36], to evaluate potential iTBS-induced changes in mood.

The last outpatient visit (V4) was a follow-up visit during which iTBS-related side effects and FMD symptoms were again assessed 24 h after receiving the last stimulation session.

### 2.3. TMS and iTBS Procedures

We delivered single-pulse TMS and iTBS, using a Magstim Rapid machine (Magstim Co., Whitland, South-West Wales, UK) and a “figure of 8” cooled butterfly coil. A single pulse TMS was used to localize the motor hotspot and determine the resting motor threshold (RMT), using the adaptive threshold hunting procedure [37]. We used an accelerated iTBS protocol, which entailed three iTBS sessions per day, with at least a 20 min interval between sessions. The duration of the inter-session interval was based on previous studies indicating that a period of ≥15 min between consecutive iTBS blocks enhances cortical plasticity [38]. Further details on the stimulation parameters, neuronavigation procedures and context of stimulation are provided in Appendix A.

Stimulation parameters: Each iTBS session consisted of 600 pulses in 50 Hz bursts of three pulses, separated by 200 ms (i.e., a 5 Hz frequency) for 2 s, followed by 8 s of no pulses over about 190 s [39]. The magnetic field intensity for each session was set at 120% of that participant’s RMT and was increased/decreased in a ramp-like fashion at the onset and offset of each iTBS session to minimize scalp discomfort/pain.

Context of stimulation: While receiving iTBS, participants wore earplugs and were seated in a comfortable chair while watching a video extracted from a nature documentary on a 15-inch computer screen placed at a distance of 60 cm. This procedure allowed for a standardized ‘context of stimulation’ between sessions and across subjects.

**Figure 2:**
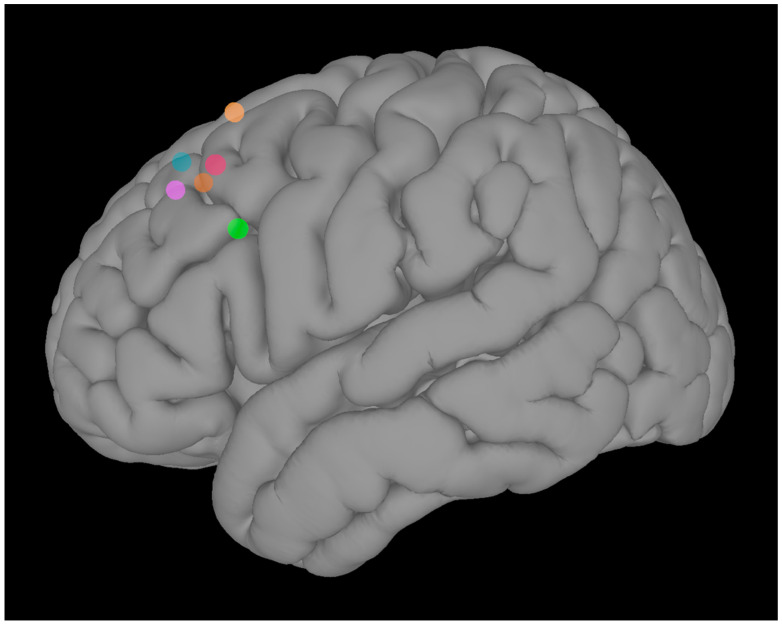
Individualized left dorsolateral prefrontal cortex targets.

Target Identification: To identify the individualized iTBS target in the left DLPFC, we used rsFC data (pre-task run) collected at V1. Pearson’s r was computed between the BOLD time series of each voxel in the left DLPFC and the average BOLD time series of the left amygdala. The coordinates of the voxel with the highest r value in the left DLPFC were identified as the iTBS target for each subject (Figure 2). This connectivity-based targeting approach offers the advantage of generating individualized stimulation sites, and allows to target remote, but functionally interconnected, brain regions. This could be particularly relevant, considering that the average clinical efficacy of left DLPFC TMS is related to intrinsic functional connectivity between the TMS target and distal brain regions [39].

Once identified, the individualized iTBS target was then marked on the individual structural MRI, using the neuronavigation software (Brainsight, Rogue Research, Inc., Montreal, QC, Canada), to ensure precise coil positioning during each session and across sessions. Further details on the neuronavigation procedure are provided in Appendix A.

### 2.4. Image Acquisition

Imaging was acquired during each visit with a 3-T MR750 GE scanner, using a 32-channel head coil. Each fMRI scan included five consecutive runs in the following order: anatomical scan (~5 min); resting state (~6 min), task (2 runs, ~10 min each), and resting state (~5 min). The full parameters are provided in the Appendix A.

### 2.5. fMRI Task and Stimuli

The processing of emotional faces was measured, using a modified version of the procedures described by Voon and colleagues [9]. Briefly, three different block conditions of fearful, happy and neutral face stimuli from Karolinska Directed Emotional Faces (70 different faces; 35 male and 35 female posing happy, fearful, and neutral expressions, for a total of 210 stimuli) were presented. The neutral face consisted of 25% happy morphed with 75% neutral, as 100% neutral faces were previously found to be aversive. Seventeen 24 s blocks (16 stimuli per block) of two runs were shown interspersed with 11 s fixation rest. The stimuli were presented centrally for 1 s with a fixation cross present for 0.5 s between trials. The images were randomized within blocks. All stimuli were back projected onto a translucent screen behind the bore of the magnet, visible via an angled mirror placed above the participant’s head. To ensure that the subjects attended the images, gender judgment button responses were acquired during the stimulus presentation period via a fiber optic button box. The facial emotion processing task is an implicit processing task; the behavioral responses were not of interest and thus not included in the analyses. The task was performed using Presentation^®^ software (Version 14.0, Neurobehavioral Systems, Inc., Berkeley, CA, USA).

### 2.6. Imaging Processing and Data Analysis

The fMRI data from each subject were processed, using AFNI (v16.2.16 [40]; afni.nimh.nih.gov). The resting-state FC data were analyzed, using the first (pre-task) run of each study visit. The left amygdala and the individual DLPFC iTBS targets were defined as regions of interest (ROIs). Pearson’s *r* was computed between the average BOLD time series of the left amygdala as defined in cytoarchitectonic atlas [41]; −23, −4, −20; 63 voxels 3 × 3 × 3) and the individual DLPFC stimulation target, which was derived using a 6-mm radius sphere region of interest (ROI) centered on the coordinates of the iTBS targets (see section below). Then, we applied *r*-to-*z* Fisher transformation and tested whether multiple sessions (‘doses’) of iTBS induced changes in the left amygdala-DLPFC target, using a mixed linear effects model. The FC between the left amygdala and the whole brain was computed in a similar manner and used as a covariate to control for individual differences in whole brain FC. Given that the left and right amygdala are highly connected, we also explored whether iTBS also exerts a neuromodulatory effect on the right amygdala-DLPFC target FC.

For the task-fMRI data, we performed a ROI-based analysis, with the left amygdala selected as the ROI, and compared the fear–happy linear contrast over study visits, using a mixed linear effects model. We repeated this analysis also to explore iTBS-induced changes in right amygdala activation patterns.

A complete description of the image preprocessing and analysis procedures, including ROI definition, is provided in Appendix A.

### 2.7. Statistical Analysis

Mixed linear effects models were used to test the effects of multiple doses of iTBS on the imaging, behavioral and clinical outcome measures collected in this study. A detailed description of the data analyses is provided in Appendix A. All statistical analyses were performed using R V.3.0.2. The level of significance for all tests was set to 0.05.

Resting-state functional connectivity (rsFC) data: The effect of the dose, or treatment accumulation over consecutive days, on the rsFC between the amygdala and the individualized iTBS target within the left DLPFC was tested, using a mixed linear effects model. In this design, the rsFC was the outcome variable, the dose was the continuous covariate of interest, the amygdala-whole brain rsFC was an additional covariate, and the patient was the random variable. The dose of the baseline visit was set to 0, while the dose of treatment visits one and two were set to 1 and 2, respectively.

Task data: The pairwise differences between V1 and the treatment visits (V2 and V3) were tested for the fear–happy linear contrast, using a linear mixed effects model. In this design, the fear–happy linear contrast Δβ value was the outcome variable, visit was the categorical variable of interest, the visit-specific negative valence score was an additional covariate, and the patient was the random variable. 

The pairwise differences between the happy–neutral and fear–neutral linear contrasts were tested for each treatment status (‘treatment’ or ‘baseline’), using paired two-sample *t*-tests. In these tests, the two groups were the two contrasts (happy–neutral and fear–neutral).

Arousal and valence ratings: The pairwise differences between the baseline visit and the treatment visits were tested for the arousal, negative valence, and positive valence scores, using three linear mixed effects models. In these designs, arousal or valence scores were the outcome variable, the visit was the categorical variable of interest, and the patient was the random variable. The effect of the iTBS dose on these scores was tested, using three linear mixed-effect models. In these designs, arousal or valence scores were the outcome variable, the dose was the continuous covariate of interest, and the patient was the random variable. The dose of the baseline visit was set to 0, while the dose of treatment visits one and two were set to 1 and 2, respectively.

Simplified-Functional Movement Disorders Rating Scale (S-FMDRS) scores: The difference between the pre-iTBS S-FMDRS score and post-iTBS score was tested, using a linear mixed effect model. In this design, the S-FMDRS score was the outcome variable, the within-visit time (pre- or post-iTBS) was the categorical variable of interest, and the patient was the random variable. Only data from V2 and V3 were used to fit this model. Post hoc paired two-sample t-tests were performed to compare pre-iTBS S-FMDRS score to the post-iTBS S-FMDRS score within each visit (V2 and V3), separately.

The pairwise differences between the pre-iTBS S-FMDRS collected at V2 and the scores collected at pre-iTBS V3 and V4 were evaluated, using a mixed linear effects model. In this design, the S-FMDRS score was the outcome variable, visit was the categorical covariate of interest, and patient was the random variable. The difference between the S-FMDRS score collected at V1 and the score collected at pre-iTBS V2 was evaluated, using a paired two-sample *t*-test.

Correlation analyses between changes in S-FMDRS scores from V4 to pre-iTBS V2 and changes in the corticolimbic rsFC and in the left amygdala response to emotional stimuli (happy–fearful facial stimuli) from V3 to V1 were analyzed, using the Pearson correlation coefficient.

## 3. Results

Study subjects (3 males and 3 females; ages 24–57 years, mean = 48.8, SD = 12.3) reported an average disease duration of 10.7 years (±7), with a baseline S-FMDRS score of 14.5 (±6.8). The abnormal movements reported included tremor and jerking movements (75%), abnormal gait/balance (65%), abnormal speech (50%), abnormal posturing/dystonia (35%). Four subjects had a lifetime history of depressive and/or anxiety disorders and only one subject had a current diagnosis of generalized anxiety disorder. Both depressive and anxiety symptom severity were in the mild range (Hamilton Depression Scale = 2.7 ± 1; Hamilton Anxiety Scale = 8 ± 9.1).

Due to motion artifacts, baseline rsFC data were not available for one subject, who was excluded from the analysis (V1–V3: *n* = 5). Task-based data were collected at all timepoints, except for one subject, who could not perform the fMRI task on V3 for an unexpected scanner shutdown (V1: *n* = 6; V2: *n* = 6; V3: *n* = 5).

### 3.1. Safety and Tolerability

All participants completed the entire study protocol without unexpected, serious adverse events. Four subjects reported a mild, transient (<60 min) headache, beginning during or shortly after the first iTBS session (Visit 2). Three subjects experienced headaches also following the second stimulation visit (Visit 3). Headaches resolved without intervention with the exception of one participant who required a single dose of acetaminophen. Two participants reported mild to moderate scalp pain after the first iTBS block. No negative side-effects in affective assessments were reported or observed after iTBS. No participant experienced any signs of mania or suicidality. The iTBS Monitoring Questionnaire revealed no seizures, fainting, difficulties in speaking or understanding speech, or impairment of thought.

### 3.2. Functional MRI

**Figure 3:**
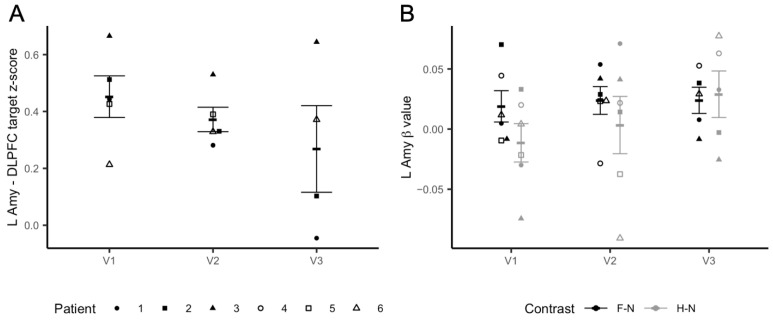
Imaging results. Panel (**A**) shows changes in rsFC between the individualized DLPFC target and the left amygdala. The y axis represents changes in z-transformed region-to-region correlation strength as a result of iTBS. Panel (**B**) shows changes in the left amygdala reactivity to fearful to neutral (F–N) vs. happy–neutral (H–N) faces during the fMRI task. The y axis represents left amygdala beta values. Error bars represent SEM.

Left Amygdala–DLPFC iTBS target resting-state functional connectivity: After two blocks of iTBS (3 daily sessions; 3600 pulses), we observed a significant iTBS dose effect on the functional connectivity between the left amygdala and the individualized iTBS DLPFC (*t* = −2.404, *p* = 0.047), which decreased from baseline (V1) to V3, as shown in Figure 3A. Exploratory analysis using the right amygdala as the ROI also found a significant effect of the dose on connectivity between this region and the iTBS target (*t* = −3.095, *p* = 0.018) (Figure S1A).

Left Amygdala Reactivity: Linear mixed models found a trend level dose effect of iTBS on left amygdala activation in response to positive–negative emotional stimuli (happy–fearful facial stimuli) (*t* = −2.182, *p* = 0.057). Specifically, at baseline (V1) there was a difference in the left amygdala response to happy vs. fearful faces (*t* = −3.518, *p* = 0.017). This difference was not significant after iTBS (V2: *t* = −0.852, *p* = 0.43; V3: *t* = 0.323, *p* = 0.76). The changes can be explained by modulations to the response to happy faces. The plot in Figure 3B shows that while the neural response to fearful vs. neutral faces remained substantially stable after both iTBS sessions, left amygdala reactivity to happy vs. neutral faces increased after receiving both iTBS blocks. Conversely, we did not find an iTBS dose effect on the right amygdala response to fearful–happy faces (*t* = −1.846; *p* = 0.0979) (Figure S1B).

### 3.3. Valence and Arousal Levels

**Figure 4:**
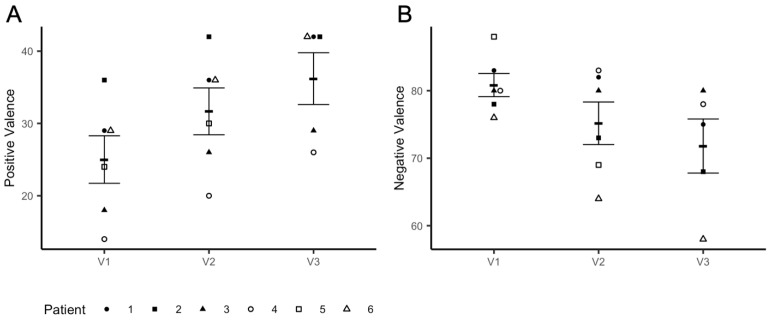
Changes in valence levels. Panel (**A**) shows an increase in positive valence ratings from Visit 1(V1) to Visit 3 (V3); panel (**B**) shows a decrease in valence ratings in response to fearful faces from V1 to V3. Participants received iTBS (3 daily session) on V2 and V3.

Consistent with the imaging results, we observed a significant iTBS dose effect on valence ratings associated with fearful faces (*t* = −3.069, *p* = 0.012), which decreased at V3 when compared to the baseline (V1–V3: *t* = −2.912, *p* = 0.017) (Figure 4A). These changes were accompanied by a marked dose effect on positive valence ratings (*t* = 10.784, *p* < 0.0001), which increased at V3 compared to the baseline (V1-V3: *t* = 11.276, *p* < 0.0001) (Figure 4B). No effects of iTBS on arousal levels in response to both positive and negative emotional stimuli were found (V1–V3: *t* = −1.738, *p* = 0.116) (data not shown).

### 3.4. Simplified-Functional Movement Disorder Rating Scale Scores

**Figure 5:**
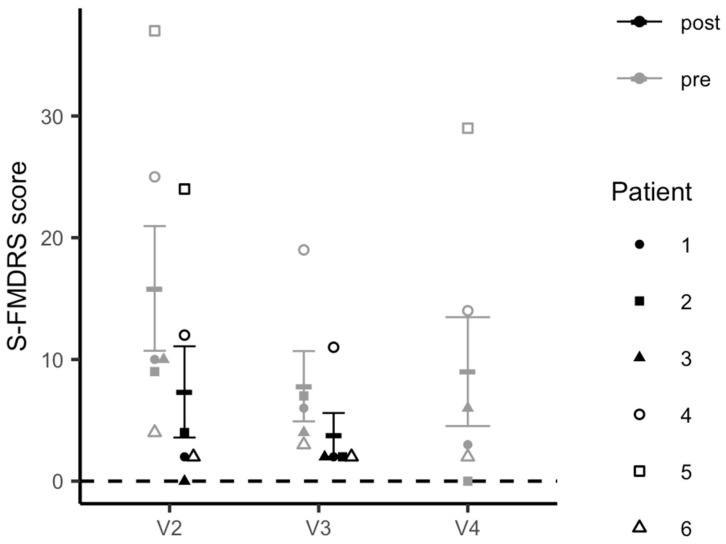
Changes in Simplified-Functional Movement Disorder Rating Scale (S-FMDRS) scores.

The linear mixed-model analysis revealed a significant decrease in FMD symptom severity from pre- to post-iTBS across both stimulation visits as measured by S-FMDRS scores (t = 5.439, *p* <.0001) (Figure 5). This decrease was significant within each stimulation visit (pre- to post-iTBS V2: *t* = 4.715, *p* = 0.0053; pre- to post-iTBS V3: *t* = 3.266, *p* = 0.0309) and between pre-iTBS scores collected at V2 and those at the follow-up visit (V4) (*t* = −5.490, *p* = 0.0004). There was no difference between S-FMDRS scores collected at V1 (baseline) and those measured at V2 prior to the stimulation session (*t* = 0.498, *p* = 0.640).

### 3.5. Correlations between Imaging Data and FMD Symptom Severity

We explored the possible relationship between changes in S-FMDRS scores and changes in rsFC between the left amygdala and the individualized iTBS DLPFC and noted a trend toward a positive correlation (r = 0.93; *p* = 0.06). We did not observe any correlation between changes in the S-FMDRS scores and changes in the amygdala response to emotional stimuli (data not shown).

## 4. Discussion

This study is, to our knowledge, the first to investigate the effects of left prefrontal iTBS on neurocircuitry, behavioral and clinical manifestations of FMD. First, we showed that an accelerated iTBS protocol was well-tolerated with a good safety profile. The most frequently reported side effect was occasional, mild headache, which remitted either spontaneously or following acetaminophen administration. Second, we found that multiples doses of iTBS targeting functionally relevant sites within the left DLPFC decreased fronto-amygdala connectivity and also influenced amygdala reactivity to emotional stimuli during an affective face perception task. These neurocircuitry changes were associated with variations in subjective indices of emotion processing, with valence levels in response to negative stimuli decreasing following iTBS. Furthermore, we also observed a significant reduction in FMD symptom severity post stimulation as measured by the S-FMDRS.

Our finding of decreased corticolimbic rsFC after iTBS parallel the results from previous studies, showing that targeted prefrontal stimulation reduced both within- and between-network connectivity [42]. Specifically, active iTBS compared to sham is shown to decrease the connectivity between DLPFC and insula [26], and between the DLPFC and nodes of the default mode network [25] in healthy volunteers. Importantly, recent research has suggested that iTBS efficacy in patients with several psychiatric disorders may depend upon ‘normalization’ of pathologically increased connectivity [27]. This mechanism may also contribute to the effects of prefrontal stimulation in FMD patients. Morris and colleagues [8] reported that individuals with FMD compared to controls exhibit elevated rsFC between amygdala and DLPFC, in line with the direction of connectivity observed at baseline in our study sample. This abnormal connectivity pattern differentiates FMD patients from those with mood and anxiety disorders, who generally show reduced connectivity between these regions. Therefore, heightened DLPFC-amygdala coupling may represent a specific brain feature of FMD, which can be targeted and remodeled via iTBS.

In addition to the effects on corticolimbic rsFC, we also observed a marginal effect of prefrontal iTBS on amygdala reactivity to affective face stimuli. Specifically, during V1 (baseline), left amygdala activation to fearful vs. neutral faces significantly differed from happy vs. neutral faces. This finding is consistent with a prior study reporting enhanced left amygdala activation to negative vs. neutral stimuli in FMD patients [10]. Importantly, the authors found that the magnitude of the amygdala response elicited by negative stimuli increased following repeated exposure. Impaired amygdala habituation to emotional stimuli has been repeatedly reported in individuals with FMD (for a review see [6]). In our study, targeted prefrontal iTBS appeared to prevent amygdala sensitization to negative stimuli, while increasing response to positive stimuli. The observed changes in amygdala connectivity and activation following stimulation are in line with findings from a recent pilot study in healthy volunteers showing that a single session of connectivity-based rTMS of the medial PFC has a downstream effect on the amygdala and corticolimbic circuitry [43].

Confirmatory behavioral analyses indicated that changes in amygdala reactivity were accompanied by a decrease in negative valence and an increase in positive valence ratings following multiple doses of iTBS, which may reflect changes in the salience attributed to affective stimuli. Together, these results parallel previous studies showing that left DLPFC stimulation improves emotion processing by inhibiting negative bias and improving affective processing of positive stimuli [14,20,21,22,23].

Conversely, our exploratory analysis did not reveal an effect of iTBS on right amygdala activation during the affective face perception task. This lack of effect could be due to the different roles that the left and right amygdalae appear to play in emotion processing as suggested by neuroimaging studies [44]. Studies in patients with FMD reported alterations in both right and left amygdalae during emotion processing, with the right amygdala showing heightened reactivity to emotional stimuli, regardless of their valence [9], and the left amygdala exhibiting both hyperactivity and sensitization to negative stimuli [10]. In the current study, we chose to modulate the left prefrontal-amygdala circuitry, given that this circuitry is directly implicated in processing aversive stimuli [45]. Such stimuli have been shown to elicit defensive behavior and modulate motor function in FMD patients [46]. Furthermore, right but not left DLPFC stimulation increases both the right amygdala response to negative stimuli as well as anxious arousal levels [23,47], which could, in turn, trigger and/or exacerbate functional motor symptoms.

This hypothesis is, in part, supported by our observation that multiple doses of iTBS induced a marked reduction in FMD symptom severity, thus suggesting a direct causal role of fronto-amygdala circuitry in FMD, as indicated by an increasing body of neuroimaging studies [6]. We also found a trend toward a positive correlation between changes in FMD symptom severity and changes in fronto-amygdala connectivity, which further supports the central role of this circuitry in FMD pathophysiology. Furthermore, this observation is in line with previous studies in depressed patients, showing that changes in connectivity underlie TMS-induced symptom improvement [27,48,49].

Our study should be interpreted in light of some limitations. First, this was an open-label study. All participants knew that they would receive active iTBS, posing the risk for a placebo effect, as for any other intervention. However, we measured the effects of left prefrontal iTBS on brain, behavioral and clinical outcomes and found an effect of iTBS on both subjective and objective measures. Second, the lack of a sham condition poses the question as to whether the observed changes are directly linked to the left prefrontal iTBS received by subjects or are related to an effect of time. Although the single-arm design of our study prevents from drawing firm conclusions, it is important to notice that the direction of changes in fronto-amygdala rsFC and in amygdala activity during the emotion processing observed in our study is in line with previous sham-controlled TMS studies [20,25], thus supporting a direct neuromodulatory effect of iTBS. Finally, further limitations to consider are the small sample size and the lack of a long-term follow-up assessment. However, as a proof of concept, our study was conducted primarily to establish the safety and feasibility of an accelerated iTBS protocol in patients with FMD as well as of our entire study procedures (e.g., connectivity-based target selection, and offline fMRI-iTBS paradigm). In addition, we also gained preliminary evidence that the prefrontal-amygdala circuitry may represent an effective target for neurocircuitry-based interventions for FMD.

Larger sham-controlled, randomized clinical trials are required to fully investigate the efficacy of this intervention, also in combination with other therapies for FMD, and to further characterize its mechanisms of action.

## Data Availability

Data are available upon request to the corresponding author.

## Supplementary Materials

The following are available online at https://www.mdpi.com/article/10.3390/brainsci11060791/s1, Figure S1: Exploratory imaging analysis results.

## Appendix A

### Appendix A.1. Exclusion/Inclusion Criteria

Exclusion criteria included comorbid neurologic diseases: lifetime history of psychosis or bipolar disorder, current diagnosis of alcohol and substance use disorders, obsessive-compulsive disorder, major depressive disorder or post-traumatic stress disorder; current suicidality; and any change in psychoactive pharmacotherapy in the 4 weeks prior to the study. Subjects were also excluded if they had contraindications to TMS administration and/or MRI, including the following: therapy with pro-convulsant medications; a history of traumatic brain injury; any personal or family history (1st degree relatives) of seizures other than febrile childhood seizures; abnormal clinical MRI brain; hearing loss; tinnitus; and pregnancy/lactation.

### Appendix A.2. Visit 1–Clinical and Behavioral Assessments

During Visit 1, all participants were screened for psychiatric diagnoses, using the Structured Clinical Interview for the Diagnostic and Statistical Manual of Mental Disorders, Edition IV, Text Revised (DSM-IV-R), Patient Edition [32]. Anxious and depressive symptomatology were assessed, using the Hamilton Anxiety Rating Scale (HAM-A) [50] and the Hamilton Rating Scale for Depression (HAM-D) [51]. Participants also completed the Profile of Mood State (POMS) [52], the Young Mania Rating Scale (YMRS) [36], the Columbia Suicide Severity Rating Scale (C-SSRS) [53] and the Symptoms Checklist 90-revised (SCL 90-R) [54]. The Childhood Trauma Questionnaire (CTQ) [55] and the Traumatic Life Events Questionnaire (TLEQ) [56] were administered to assess exposure to stressful events during early life and adulthood. To establish contraindications to MRI and TMS administration, we administered two internally generated tools: the MRI safety screen questionnaire and the TMS safety screen questionnaire.

### Appendix A.3. Arousal and Valence Rating Procedures

Subjects were asked to view on a computer screen a series of 18 pictures (6 fearful, 6 happy and 6 neutral faces) selected from the Karolinska Directed Emotional Faces dataset [57]. Pictures were presented in a random sequence, varying the order of the stimulus across the participants, with no particular stimulus occurring more than once. After each presentation of the stimulus (stimulus presentation duration  =  10 s), subjects provided their ratings on a 7-point, paper-and-pencil version of the self-assessment manikin (SAM) [33] while no longer viewing the image. To prevent inter-session habituation to such stimuli, we composed three different series of 18 pictures each, and each set of stimuli was shown at each study visit. Six alternate versions of these three stimulus sets were counterbalanced across subjects.

### Appendix A.4. MRI Procedures

Image acquisition: Imaging was acquired during each visit with a 3-T MR750 GE scanner, using a 32-channel head coil. Structural images and structural scans were collected, using a T1-weighted anatomical MRI (multi-echo magnetization-prepared rapid gradient echo [MEMPRAGE], voxel size 1 × 1 × 1 mm; repetition time [TR] 400 ms; echo time [TE] 1.69 ms; echo spacing 9.8 ms; number of echoes 4; bandwidth 650 Hz/Px; inversion time 1,100 ms; flip angle 7°; acceleration factor 2; matrix size 176 × 256 × 256; field of view [FoV] 256 mm, acquisition time: 6 min 2 s), while functional scans were acquired using a T2-weighted EPI sequence (voxel size 3 × 3 × 3 mm; number of slices 36; matrix size 36 × 72 × 72; TR 2500 ms; number of TRs 144 (resting state)/240 (task); TE 14.5 ms; echo spacing 17.8 ms; number of echoes 3). During resting state runs, participants were instructed to lie as still as possible in the scanner with their eyes closed, not to think about anything in particular and not fall asleep.

Image preprocessing: Image preprocessing and analysis was completed, using AFNI software [40]. The MPRAGE for each visit was skull stripped and nonlinearly registered to the MNI ICBM 152 2009 template using the @SSwarper function. Each functional sequence was preprocessed, using afni_proc.py with the following processing steps: despiking, slice-time correction, alignment to the processed MPRAGE*, registration to the MNI template space using the warps computed by @SSwarper*, co-registration of each functional volume*, multi-echo independent component analysis (via tedana.py), convolution with a 4-mm full-width at half-maximum Gaussian kernel, scaling each voxel time series to a mean of 100, and regression out of motion parameters. All spatial transformations (*) were concatenated and applied in a single step.

Volumes with excess motion (frame-wise displacement >0.3 mm) and/or with BOLD signal outliers in many voxels (proportion of voxels >0.1) were censored.

Region of interests: We selected three regions of interest (ROI), including the left amygdala, right amygdala and individualized iTBS sites within the left DLPFC. For the right and left amygdala, ROIs were defined using a cytoarchitectonic atlas [41]. For each amygdala ROI, the probability maps of the amygdala subdivisions were added together to form a probability map of the whole amygdala, which was threshold to a value of 0.5 to form the ROI. The DLPFC ROI was defined using a functional atlas that identified regions based on whole-brain resting-state functional connectivity [58].

Task data analysis: In order to compare task-related activation across conditions (happy, neutral, fearful), multiple linear regression was performed on the preprocessed task data, using 3dDeconvolve within afni_proc.py. Regressors for each condition were created via convolution of the stimulus onset time series with the canonical hemodynamic response function. Stimuli were considered instantaneous for this analysis. Regression yielded condition-specific β-coefficient maps, which indicated task-related activation within each voxel. Linear contrast (Δβ) maps, which indicated the difference in activation between each pair of conditions within each voxel, were also computed.

### Appendix A.5. Intermittent TBS Procedures

Individualized iTBS target neuronavigation: In order to allow for the use of standardized coordinates to mark the personalized iTBS target on the individual structural MRI within the Brainsight neuronavigation software, the processed MPRAGE was linearly registered to the MNI ICBM 152 2009 template. A linear transformation was used to conserve individual-specific spatial features, which were necessary to perform accurate landmark-based calibration of the coil prior to the iTBS session. This transformation was applied to the processed resting state sequence. Neuronavigation (Brainsight, Rogue Research, Inc., Montreal, QC, Canada) with an optical tracking system (“Polaris Vicra”, Nothern Digital Inc., Waterloo, ON, Canada) was used to ensure precise positioning of the coil over the individualized stimulation target within each session, across sessions, and across participants.
