# Supplementary figures and images for: Corticolimbic Modulation via Intermittent Theta Burst Stimulation as a Novel Treatment for Functional Movement Disorder: A Proof-of-Concept Study
Source: Brain Sci. 2021 Jun 15;11(6):791. doi: 10.3390/brainsci11060791 (PMC8232716; doi:10.3390/brainsci11060791)

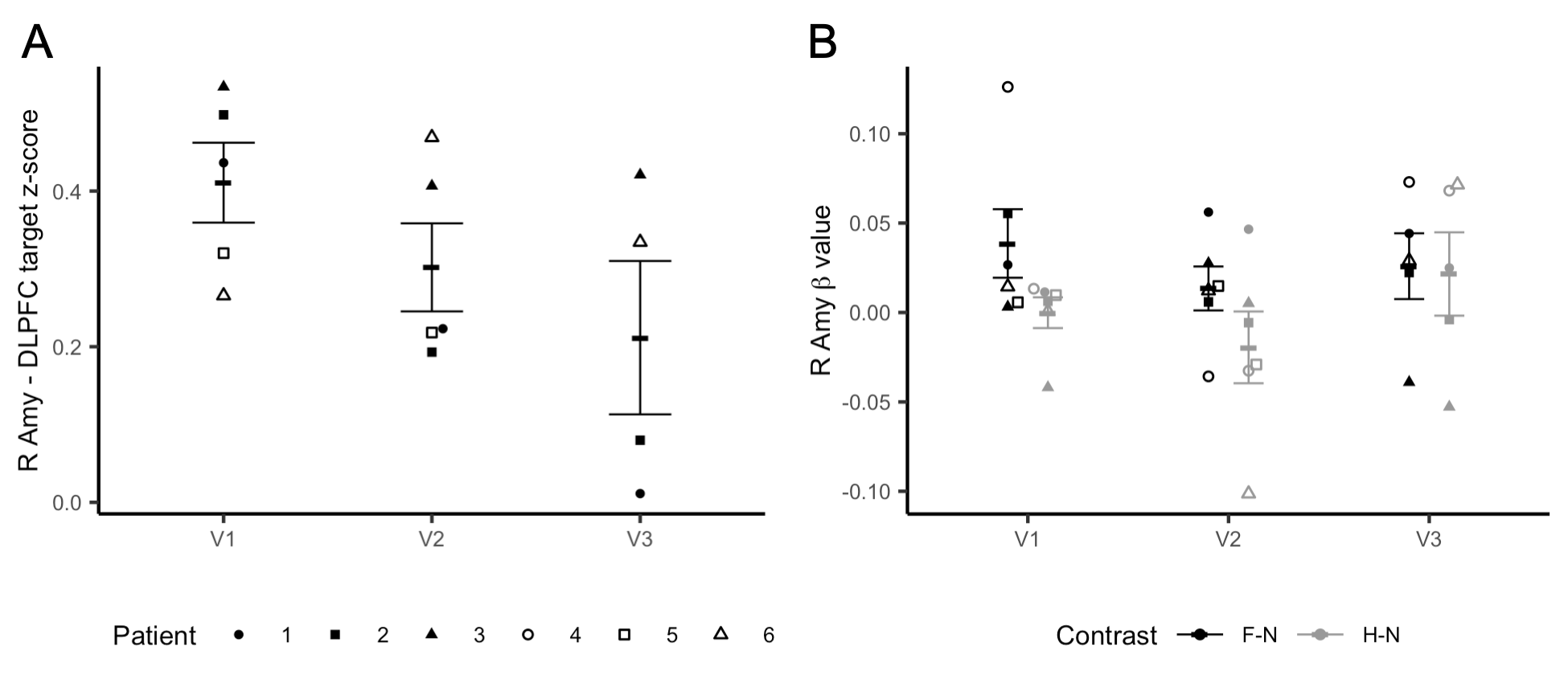

Supplement: Supplementary file 1 [file brainsci-11-00791-s001.zip › Figure S1.png]
